# Multidisciplinary team meetings for patients with complex extremity defects: a retrospective analysis of treatment recommendations and prognostic factors for non-implementation

**DOI:** 10.1186/s12893-021-01169-4

**Published:** 2021-03-29

**Authors:** Dimitra Kotsougiani-Fischer, Sebastian Fischer, Jan Warszawski, Paul A. Gruetzner, Gregor Reiter, Christoph Hirche, Ulrich Kneser

**Affiliations:** 1 Department of Hand, Plastic and Reconstructive Surgery, Microsurgery, Burn Center–BG Trauma Center Ludwigshafen, Hand and Plastic Surgery of the University of Heidelberg, Ludwig Guttmann Str. 13, 67071 Ludwigshafen, Germany; 2 Department of Trauma and Orthopedic Surgery, BG Trauma Center Ludwigshafen, Ludwig Gutmann Strasse 13, Ludwigshafen, 67071 Germany

**Keywords:** Multidisciplinary team meetings, Complex extremity defects, Free flap, Bone reconstruction

## Abstract

**Background:**

This study aimed to assess a multidisciplinary team (MDT) meeting approach for the management of patients with complex extremity defects, analyze treatment recommendations, and evaluate factors influencing non-implementation.

**Methods:**

All patients introduced to an MDT meeting for complex extremity defects from 2015 to 2017 were included in a retrospective cohort study. Patients’ characteristics and defect causes were evaluated. Treatment recommendations (TR) of MDT meetings and subsequent implementation were reviewed (cohort with implementation of TR versus cohort with non-implementation of TR), and factors associated with non-adherence to recommendations were statistically analyzed using logistic regression.

**Results:**

Fifty-one patients (41 male) with a mean age of 54 years were presented in 27 MDT meetings. Most of the patients (70%) suffered from reconstructive challenging or combined bone- and soft tissue defects, primarily located at the lower extremity (88%). Large skeletal defects, chronic osteomyelitis, and multi-fragmented fractures were present in 65% of cases. Forty-five percent of the patients suffered from peripheral vascular disease, necessitating surgical optimization. Of the 51 MDT decisions, 40 were implemented (78%; (32/40) limb salvage versus 22%; (8/40) limb amputation). Limb salvage was successfully achieved in 91% (29/32) of the cases. Failed limb salvages were due to flap failure (33%; 1/3), recurring periprosthetic joint infections (66%; 2/3) and concomitant reconstructive failure. Patients who underwent limb amputation, as recommended, showed proper stump healing and regained mobility with a prosthesis. Overall the MDT treatment plan was effective in 92.5% (37/40) of the patients, who adhered to the MDT treatment recommendation. In eleven patients (22%; 11/51), the MDT treatment was not implemented. MDT decisions were less likely to be implemented, if amputation was recommended (p = 0.029).

**Conclusions:**

MDT meetings represent a valid tool to formulate individualized treatment plans, avoiding limb amputation in most patients with severe extremity defects. Recommendation for limb amputation is less likely to be implemented than plans for limb salvage.

*Trial registration:* Retrospectively registered

## Background

In the past decades, multidisciplinary and innovative therapeutic concepts have revolutionized the treatment of complex extremity defects, thus successfully averting limb amputation in many cases. Depending on the defect size and localization, involvement of tissue components, and presence of infection, the reconstructive approach can range from simple to complex procedures. Large osseous defects may necessitate cryopreserved allogeneic or microvascular autologous bone transplants, bone prostheses, or bone transport via Masquelet technique [1–3]. Concomitant or extensive soft tissue defects may require microvascular free flap coverage and severe damage to muscle groups may indicate complex tendon transfer or muscle neurotization [4–6].

In addition, depending on the underlying disease, optimization of the vascular status might be necessary. Finally, patient-related factors such as comorbidities, patient preferences, and functional status influence the treatment plan, making a multidisciplinary team approach mandatory in the successful treatment of complex extremity defects [7, 8].

Multidisciplinary team (MDT) meetings are well-established for treatment recommendations in patients with cancer. They serve as a platform to provide expert reviews on patient cases, according to clinical practice guidelines and beyond, ensuring well-coordinated and multi-professional patient care [9]. Similarly, regularly scheduled MDT meetings, comprising professionals from trauma and orthopedic, plastic-, and vascular surgery, can formulate individualized treatment recommendations for patients with complex extremity defects. Thus, in 2015, an MDT meeting for patients with complex extremity defects—the so-called extremity board—was established in our unit. However, MDT meetings require significant time resources and finances. Thus, it is reasonable to question their impact on patient outcomes and evaluate their quality. An excellent method to analyze MDT performance is to assess implementation of the formulated treatment plan along with the clinical outcome. Therefore, this study aimed to investigate the implementation of treatment decisions reached in the MDT meeting for patients with complex extremity defects and determine factors associated with non-implementation of the recommendations. The secondary aim was to evaluate all implemented board decisions' clinical outcome as a criterion for the quality of the reached multidisciplinary treatment decision.

## Patients and methods

### MDT meetings for patients with complex extremity defects

**Fig. 1:**
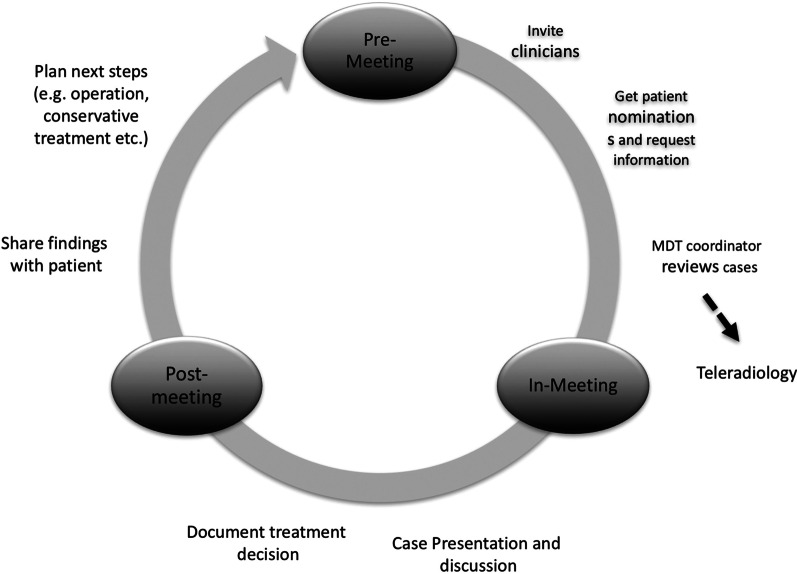
MDT meeting for complex extremity defects. Schematic drawing of the workflow of an MDT meeting for complex extremity defects

MDT meetings were held monthly at the facility of the senior author. Meetings were certified for continuous medical education credit points and announced biannually in print and via email to physicians of surrounding hospitals and private practice. The MDT consisted of trauma, orthopedic, plastic and vascular surgeons. Professionals of other disciplines (rehabilitation therapy, radiology, critical care, geriatrics) were invited if indicated. The treating physician submitted cases for presentation to the coordinator of the MDT meeting (first author DK) for review and acceptance. Submissions included a check-list that ensured medical history, relevant images (X-ray, computed tomography, magnetic resonance imaging, angiography), clinical findings and microbiological results were available [10]. Registration of cases ended two days before the next meeting to provide sufficient time for acquiring further documents or invitation of professionals of other disciplines. In the case of vascular pathologies, all relevant angiographies were transferred to a vascular surgeon before the upcoming meeting with means of teleradiology. During the MDT meeting, patient cases were presented summarizing all relevant clinical data and demonstrating all relevant imaging. After the MDT meeting, the reached treatment decision was documented in the electronic patient file. The treating physician informed the patient of the treatment recommendation. Figure 1 displays the workflow of the MDT meetings for patients with complex extremity defects.

### Study design

The local ethics committee of Rhineland palatinate (no. 2020-15004) Mainz Germany approved the retrospective study, which was designed in accordance with the ethical standards laid down in the Declaration of Helsinki and its later amendments. Since the study protocol included only a retrospective, epidemiologic evaluation of anonymized and routine patient data, informed consent was waived by the local ethics committee of Rhineland palatinate.

All medical records from patient cases presented in the MDT meetings for complex extremity defects between September 2015 and December 2017 were retrospectively studied. Epidemiologic data, such as age, gender, comorbidities, American Society of Anesthesiologists (ASA) physical status classification system, defect localization, extent of defect (bone, soft tissue, combined bone and soft tissue defect) and vascular status at the time of the MDT meeting were identified. Furthermore, MDT treatment decision and implementation were evaluated and classified as implemented (MDT treatment decision and treatment received were the same) or as non-implemented (MDT decision differed from final MDT treatment). Furthermore, reasons for non-implementation of MDT treatment decisions were gathered (patient’s preference, comorbid conditions, or new clinical information). Last, but not least, the clinical course of all patients who adhered to the MDT treatment recommendation was analyzed. This included stump healing and regained mobility in patients with limb amputation, as well as the success or failure of the surgical intervention in patients with limb reconstruction.

### Statistical methods

Data are presented as frequencies (percentages) for the categorical variables and means—standard deviation (SD) for the continuous variables. A multivariable logistic regression model was performed to identify factors associated with the non-implementation of MDT treatment decisions. Hosmer/Lemeshow (HL) tests for calibration were computed to assess the goodness of fit. The odds ratios (OR) with their corresponding 95% confidence intervals (CIs) were calculated. Statistical significance was defined as p < 0.05. Data were analyzed using the GraphPad Prism version 8.4.0 for MAC (GraphPad Software San Diego, CA).

## Results

### Demographic data

During the study period, 27 MDT meetings were held, in which 51 patients with complex extremity defects were presented. Representatives of trauma surgery and orthopedics, as well as plastic surgery, were present in all MDT meetings. Vascular surgeons were present in 19.6% (10/51) of cases. In the remaining 80.4% (41/51) of the cases, telemedicine consultations were performed with vascular surgeons. Forty-one patients were male and ten female. The mean age was 54 years, ranging from 21 to 81 years. Twenty percent of the presented patients were active smokers and 43.1% suffered from hypertension. Furthermore, diabetes mellitus was present in 23.5% of the patients.

The majority of patients had lower limb defects (88.2%, 45/51). Upper extremity defects were less common (11.8%, 6/51). Thirty-six patients (70.6%, 36/51) had combined bone and soft tissue defects. Isolated soft tissue defects occurred in 22% of cases (11/51) and isolated bone defects in 8% of cases (4/51). Of the 40 patients with bone defects (combined + isolated), segmental bone defects (defect size > 2 cm) were present in 27.5% of patients (11/40), complex multi-fragmented fractures in 27.5% (11/40) and osteomyelitis in 27.5% (11/40). Furthermore, periprosthetic joint infections were apparent in three patients (5.9%). The majority of defects were due to acute trauma (56.9%, 29/51). Rarely, peripheral vascular disease (7.8%, 4/51), tumors (3.9%, 2/51), or infection (30%, 1/51) were responsible for the extremity defect. Almost one third of patients (27.5%, 14/51) had previously received a peripheral vascular surgery and 17.6% (9/51) had pathologies in angiography at the time of the MDT meeting. Epidemiologic data are presented in Table 1.

**Table 1:** Epidemiologic data

Variables	Patients n = 51
Age, M (SD), years	53.9 ± 17.2
Gender, male, N (%)	41 (80.4%)
ASA physical health status, M (SD)	2.2 ± 0.8
Smoking history, N (%)	10 (19.6%)
Hypertension, N (%)	22 (43.1%)
Diabetes mellitus, N (%)	12 (23.5%)
Defect localization	
Upper extremity, N (%)	6 (11.8%)
Lower extremity, N (%)	45 (88.2%)
Extent of defect	
Isolated bone, N (%)	8 (4%)
Isolated soft tissue, N (%)	22 (11%)
Combined bone and soft tissue, N (%)	36 (70.6%)
Classification of bone defects	
Large segmental bone defects (defect size > 2 cm), N (%)	11 (21.6%)
Complex multi-fragmented fractures, N (%)	11 (21.6%)
Osteomyelitis, N (%)	11 (21.6%)
Periprosthetic joint infection, N (%)	3 (5.9%)
Defect cause	
Peripheral vascular disease, N (%)	4 (7.8%)
Tumors, N (%)	2 (3.9%)
Infection, N (%)	16 (31.4%)
Acute trauma, N (%)	29 (56.9%)
Previous vascular surgery, N (%)	14 (27.5%)
Pathologic vascular status at MDT meeting, N (%)	9 (17.6%)

*M* mean, *SD* standard deviation

### Outcomes

The follow-up amounted to 47.1 ± 8.6 months. Agreement in MDT treatment decisions was reached in all presented cases. Limb salvage was recommended for 37/51 (72.5%) patients, and limb amputation for 14/51 (27.5%) patients.

In over half of the patients (56.9%), a plastic surgery intervention was recommended, most commonly with free flaps (51%). When free flaps were necessary to close the defect, complex chimeric flaps were predominantly recommended, such as the combined parascapular and latissimus dorsi free flap. In over 20% of the patients, a vascular intervention or additional radiologic analyses were proposed. Furthermore, in 39.2% of patients, complex bone reconstructive options were recommended. These included avascular and vascularized bone grafting and staged procedures, such as antibiotic spacer implantation and distraction osteogenesis. MDT treatment decisions for limb reconstruction are presented in detail in Table 2.

**Table 2:** MDT treatment recommendations for limb preservation

Variables	Patients n = 51
*Vascular surgery intervention*	11 (21.6%)
Arteriovenous loop	2 (3.9%)
Percutaneous transluminal angioplasty	1 (2%)
Angiography	4 (7.8%)
Vessel-extension by vein graft	2 (3.9%)
*Plastic surgery intervention*	29 (56.9%)
Free flaps	26 (51.0%)
Chimeric parascapular and latissimus flap	8 (15.7%)
Free groin flap	1 (2.0%)
Free iliac crest flap	1 (2.0%)
Latissimus dorsi flap	7 (13.7%)
Anterior lateral thigh flap	4 (7.8%)
Parascapular flap	2 (3.9%)
Rectus abdominis flap	1 (2.0%)
Free fibular flap	2 (3.9%)
Pedicled flap	3 (5.9%)
*Trauma surgery intervention*	20 (39.2%)
Osteosynthesis	10 (19.6%)
Antibiotic spacer removal	1 (2.0%)
Antibiotic spacer implantation	3 (5.9%)
Bone debridement	7 (13.7%)
Arthrodesis	4 (13.7%)
Endoprosthesis	1 (2.0%)
Resection arthroplasty	3 (5.9%)
Bioactive glass scaffold implantation	1 (2.0%)
Autogenous Bone grafting	3 (5.9%)
Allogeneic Bone grafting	1 (2%)
Implant removal	7 (13.7%)
*Other intervention*	5 (9.8%)
Intraoperative demonstration	1 (2%)
Home vacuum therapy	4 (7.8%)

*MDT* multidisciplinary team

Overall, 78.4% (40/51) of the MDT treatment decisions were implemented. In the remaining cases (21.6%; 11/51), in which MDT treatment decision was not implemented, the main reason for discordance was the patient’s preference (6/51, 11.8%); in these cases patients chose limb salvage or no treatment instead of the recommended lower leg amputation. Further reasons for non-implementation of MDT recommendations included comorbidities (2/51, 3.9%) and new clinical information (3/51, 5.9%), which were not available at the time of MDT meeting, made limb salvage impossible, and led to discordance to the MDT treatment recommendation (Tables 3 and 4). Underestimation of the severity of peripheral vascular disease as well as progression and detoriation of the wound status made limb preservation in most of the cases impossible (Table 4).

**Table 3:** Reasons for non-implementation of MDT treatment decisions

Variables	Patients n = 51
Patient’s preference, N (%)	6 (11.8%)
Comorbidities, N (%)	2 (3.9%)
New clinical information, N (%)	3 (5.9%)

**Table 4:** Conversion of MDT treatment recommendations

Reason for non-implementation	MDT treatment recommendation	Final course of action
Patient’s preference	Implant removal ankle, free flap reconstruction lower leg	Implant removal, conservative wound treatment, shock wave therapy
Patient’s preference	Lower leg amputation	Conservative wound treatment, negative pressure therapy
Patient’s preference	Lower leg amputation	Conservative wound treatment
Patient’s preference	Lower leg amputation	Ilizarov bone transport
Patient’s preference	Free flap reconstruction, ankle arthrodesis with bone grafting	Free flap reconstruction, simple osteosynthesis
Patient’s preference	Lower leg amputation	Conservative wound treatment, negative pressure therapy
Comorbidity (severe PVD)	AV-Loop, free flap reconstruction lower leg	Iliac artery angioplasty, femoral artery angioplasty, PTA A. poplitea, lower leg amputation, prosthesis
Comorbidity (severe PVD)	Vascular imaging, free flap reconstruction, lower limb amputation	Transfemoral amputation
New clinical information (insufficient perfused soft tissue and muscles)	Humerus reconstruction by fibular free flap reconstruction, plate osteosynthesis, radial artery reconstruction, fasciocutaneous free flap reconstruction, median nerve reconstruction through sural nerve grafting	Upper limb amputation, prosthesis
New clinical information (new inguinal wound healing problem)	Femur-removal, lower leg amputation, reconstruction with osteomyocutaneous turn-up plasty as described by Sauerbruch	Femur-removal, lower leg amputation, reconstruction with osteomyocutaneous turn-up plasty as described by Sauerbruch, pedicled flap reconstruction
Comorbidity (severe PVD)	Vascular imaging, Free flap reconstruction, lower limb amputation	Transfemoral amputation
New clinical information (soft tissue of forefoot viable)	Forefoot amputation, chimeric free flap reconstruction of foot	Osteosynthesis, free flap reconstruction of the feet, skin grafting

*MDT* multidisciplinary team, *PVD* peripheral vascular disease, *PTA* percutaneous transluminal angioplasty

Multiple logistic regression analysis exploring factors that might influence the implementation of the MDT meeting decision showed that amputation intent was an important reason not to follow the recommendation with sixfold greater odds (CI 1.4–29.4; p = 0.021). Gender, age, and physical status (ASA-classification) did not influence the implementation of MDT treatment decisions (Table 5).

**Table 5:** Prognostic risk factors for non-implementation of MDT treatment decision

Variables	Odds ratio	(95% CI)	*p*-value
Gender			
Female	0.2	(0.1–2.1)	0.234
Age			
≥ 65	0.6	(0.1–3.1)	0.530
ASA-classification			
ASA 3–4	1.5	(0.3–7.6)	0.656
Treatment intent			
Amputation	6.0	(1.4–29.4)	0.021*

*MDT* multidisciplinary team, *ASA* American Society of Anaesthesiologists

*Significant, p < 0.05

To evaluate the quality of the MDT treatment recommendations, the final clinical outcome of all patients, who adhered to the MDT treatment recommendation (78.4%; 40/51), was followed. Limb salvage was recommended for 80% (32/40) of the patients and successfully achieved in 90.6% (29/32) of the cases. Failed limb salvages were due to flap failure (33%; 1/3), recurring periprosthetic joint infections (66%; 2/3), and concomitant reconstructive failure. Of note, in all patients with failed limb salvage, vascular interventions had been performed (one patient with arteriovenous loop and two patients with vessel extensions by vein grafts). All three patients with failed extremity reconstructions were debriefed in the following MDT meeting for extremity defects. Patients with amputations (20%; 8/40) showed proper stump healing and regained mobility with a prosthesis. Overall, the MDT treatment plan was effective in 92.5% (37/40) of the patients, who adhered to the MDT treatment recommendation.

Furthermore, we followed the clinical outcome of all patients who did not adhere to the MDT treatment recommendation due to patients’ preference (6/11). Sixty-seven percent of the patients (4/6) chose a conservative wound therapy with negative pressure wound therapy instead of limb amputation. Two patients were lost to follow up, and the remaining two showed persistent symptoms with pain due to pseudarthrosis and persistent open wounds. The two patients who received complex reconstructions (ilizarov bone transport and free flap reconstruction) healed uneventfully and regained their mobility.

### Case presentation

**Fig. 2:**
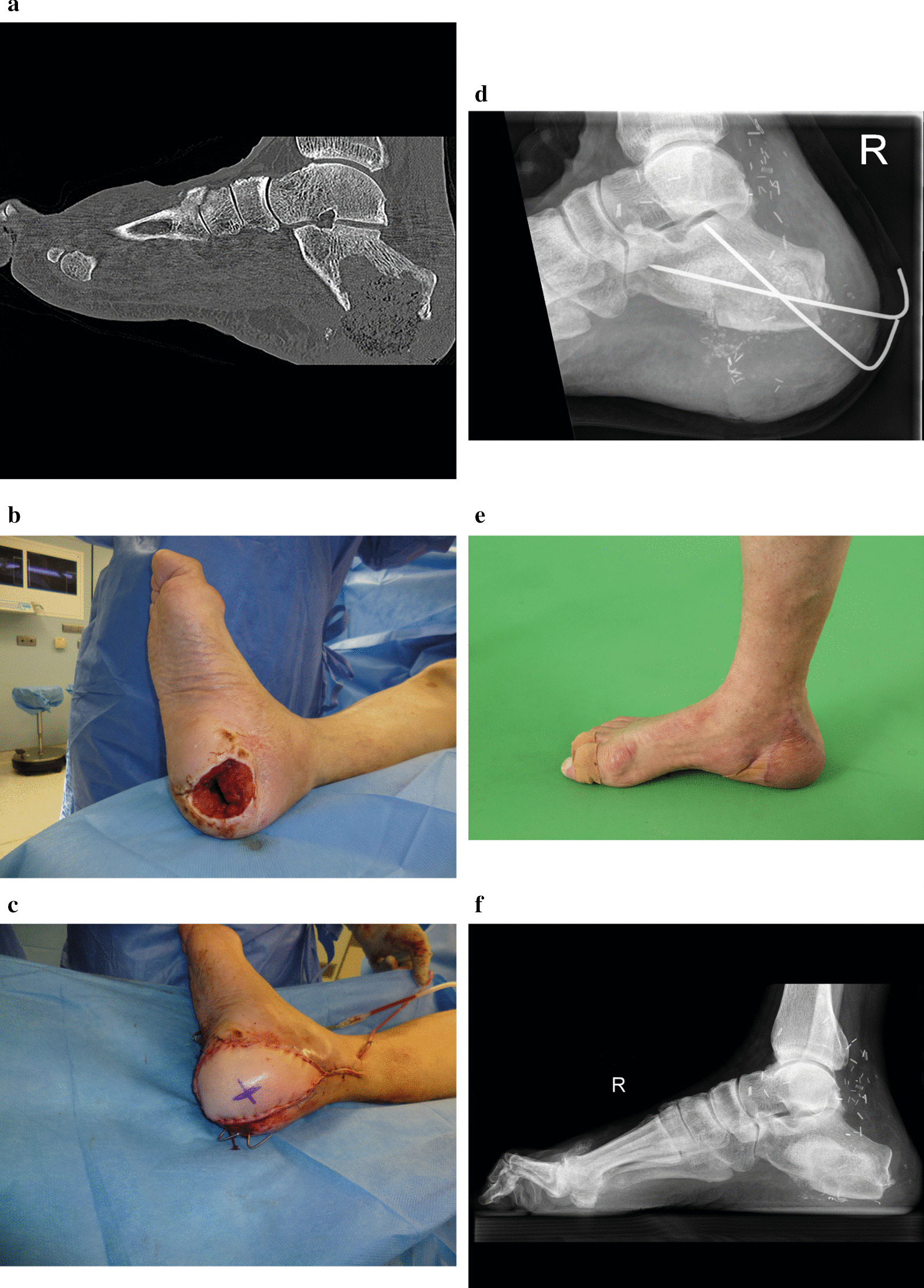
Limb-threatening chronic osteomyelitis of the calcaneus with subtotal resorption of the calcaneus and concomitant heel ulcers in a 66-year-old male patient. **a** Computed tomographic imaging of the right foot with subtotal bone resorption of the calcaneus due to chronic osteomyelitis. **b** Intraoperative lateral view of the right lower leg and foot with a major soft-tissue defect in the right weight-bearing heel before reconstruction. **c** Postoperative lateral view of the reconstructed heel. The bone defect has been reconstructed with a free vascularized iliac crest flap and the soft-tissue defect with a free groin flap. In-flap anastomoses were performed between the nutrient vessels of the iliac crest and groin flap and further anastomoses performed to the A. tibialis posterior. Furthermore, 15 cc bioactive glass scaffold implantation was performed. **d** X-ray of the right foot 21 days after internal fixation of the iliac crest bone segment by two k-wires. **e** + **f** Longterm follow-up after 1 year showing full weight-bearing and sufficient consolidated right calcaneus in the X-ray

A 66-year-old male patient presented with a chronic open wound of the calcaneus after multiple Achilles tendon lesions. We performed computed tomographic imaging of the right foot, which showed subtotal bone resorption of the calcaneus due to chronic osteomyelitis (Fig. 2a). Multiple osseous and soft tissue debridements with the application of negative pressure wound therapy followed, which showed a significant soft-tissue defect of 7 cm diameter in the right weight-bearing heel and a major osseous defect (Fig. 2b). Microbiological analysis of soft tissue and bone samples showed Staphylococcus haemolyticus and Citrobacter amalonaticus. The patient was put on meropenem and fosfomycin therapy for six weeks. The bone defect was reconstructed with a free vascularized iliac crest flap. Wound coverage was achieved with a free groin flap (Fig. 2c). In-flap anastomoses were performed between the nutrient vessels of the iliac crest and groin flap, and further anastomoses were performed to the A. tibialis posterior. Furthermore, a 15 cc bioactive glass scaffold was implanted. The postoperative course was uneventful. X-ray analyses 21 days after internal fixation of the iliac crest bone segment by two k-wires showed a good iliac crest position (Fig. 2d). In the longterm, the patient showed full weight-bearing after one year (Fig. 2e). Furthermore, X-ray analysis revealed a sufficient consolidated right calcaneus (Fig. 2f).

## Discussion

This consecutive series of treatment recommendations made within an MDT meeting for patients with complex extremity defects found that the formulated treatment plan was effective in 92.5% of the patients who adhered to the MDT treatment recommendation. Limb salvage was recommended with a complex reconstructive procedure for 72.5% of the patients and limb amputation in 27.5% of the cases. However, in 21.6% of the cases the MDT treatment decision was not implemented and a discordance to the initial MDT treatment recommendation detected. Of the decisions that changed after the meeting, the most common reason was that it was unacceptable to the patient. In particular, the decision to amputate an extremity was significantly associated with the non-implementation of the MDT treatment plan.

MDT meetings are nowadays mandatory for treatment decisions in patients with malignancies. Interestingly, recent studies show that a discordance from the initial tumor board decision in patients with breast cancer is found in 8% of cases [11]. In patients with colorectal or gastrointestinal tumor, the therapy can differ in as much as 10% to 40% cases from the initial tumor board decision [12]. It should be borne in mind that there are better data and evidence in oncology and well-developed treatment guidelines. In contrast, no specific guidelines exist for the treatment of devastating or composite soft-tissue and bone defects. Each case is unique and necessitates an individualized therapeutic approach. We detected a moderate rate of discordance (21.6%) between the initial MDT treatment decision and final treatment plan. The key reason for non-implementation of MDT recommendations in 11.8% of the cases was that the MDT treatment decision was not acceptable to the patient. Final treatments were more conservative than initially planned, choosing no therapy or limb reconstruction instead of amputation. Similarly, in oncology, the patient’s preference is one of the most important factors for a discordance between the treatment decision of the MDT meeting and final clinical outcome [13]. An option to increase patient’s adherence to the MDT treatment plan may be the involvement of patients in the treatment decision process by including them in the MDT meeting. This topic has caused controversy in several previous studies focusing on multidisciplinary breast cancer conferences [14, 15]. However, research on the risks and benefits of patient participation has not provided substantiated findings yet [14]. Furthermore, Hamilton et al. showed recently that MDT decision-making process needs a substantial review if patients are to be effectively involved [16].

Further reasons for non-implementation of treatment plans were comorbidities (3.9%) and new clinical information (5.9%), which made it impossible to implement the board’s decision, including periprocedural risk factors. This is an important finding, which has to be addressed in order to improve the quality of the MDT extremity board. In particular since underestimation of the severity of the peripheral vascular disease or progress and detoriation of the wound status made free flap reconstruction and limb preservation impossible.

However, the lack of consideration of the patient’s comorbidities during multidisciplinary tumor conference decision-making has also been demonstrated in previous studies [13]. One strategy to improve MDT meetings’ structural quality is to use checklists for patient case preparations [17]. Although our MDT meeting checklist tool did include a section for patient’s comorbidities, new clinical information or worsening of a clinical diagnosis may inhibit the implementation of a treatment decision.

This study also identified factors associated with the non-implementation of the MDT treatment plan. As expected, the MDT treatment decision for limb-amputation was a significant factor in not adhering to the treatment decision (p = 0.029). Patients preferred to get no therapy or insisted on the attempt of complex reconstructive procedures in the view of a rather low chance of success instead of limb amputation. However, postoperative functionality and survival benefit were critical factors in the decision-making process [18, 19]. Thorough patient education is urgently needed to increase therapy adherence. Furthermore, debriefing of changed therapeutic plans is necessary when the MDT treatment decision is not followed.

The concept of MDT meetings for patients with complex extremity defects is still relatively new [10]. In 2015 we established these MDT meetings comprising experts from orthopedic and trauma, plastic and vascular surgery analogous to multidisciplinary tumor board meetings. The aim was to find a treatment plan for challenging cases, in which patients presented with complex defects of the extremities, involving at least two surgical disciplines for limb salvage (Fig. 2). Furthermore, patients with limb-threatening diseases in whom the feasibility and sense of limb preservation was insecure were presented. Of note, approximately 300 free flap extremity reconstructions are performed annually in our clinic, of which only 5–6% were presented in the MDT meeting for extremity defects. Patients with simple soft tissue defects of the extremities, necessitating all kinds of free flaps (fasciocutaenous-, musculocutaneous-, osseous- or composite free flaps), were not presented in the MDT meeting.

The complexity and severity of the cases presented in the MDT meeting are further reflected by the high number of patients with combined bone- and soft tissue defects (70%) as well as extensive skeletal defects, multi-fragmented fractures, or infected bone (64.7%). Furthermore, 45.1% of the patients had a pathologic vascular status, complicating the therapy.

Although 70.6% of the patients from this study suffered from extensive, combined bone and soft tissue defects, the decision to restore the extremity was reached in almost two-thirds of the patients. When the MDT treatment plan for limb reconstruction was followed, limb salvage with proper weight-bearing was achieved in 91% of cases. This high percentage of weight-bearing patients is quite satisfying, taking into consideration the severity of limb-threatening diseases. In patients, in which limb amputation was the only viable option, the MDT meeting provided a platform to define the optimal limb length. Our outcome analysis showed that patients with recommended limb amputation showed a proper stump healing and regained the ability to walk with prosthesis. Telemedicine was used in quite a high percentage of patients (80%) to ensure all participating disciplines' input and presence for effective decision-making. In this context, a recent study from Endean et al. showed that telemedicine evaluation of patients with vascular pathologies is accurate and effective, and compares to on-site evaluations [20].

The requirements for a functioning MDT meeting for patients with complex extremity defects were comparable to those of cancer MDT meetings [13, 17]. The MDT meeting had a sensible team structure and was managed by an integratively-acting expert in extremity reconstruction. Structured presentations were adapted to standardized checklists, including complete diagnosis, as well as patient-related factors such as psychosocial factors and patient preferences regarding the treatment recommendation. For the MDT treatment decision, an expert committee consisting of decision-makers from all surgical disciplines was present. Auditing of non-implementation of MDT treatment decisions and studying reasons for changed decisions provided us useful feedback. To maximize MDT meeting performance and to achieve patient-centered decisions, patient’s preferences, and complete patient profiles should be provided in every patient presentation.

This study has some limitations. Although this is the first study of MDT treatment implementations in patients with complex extremity defects, the main limitation of this study stem from its retrospective observational study design -specifically, the potential for confounders, lack of clinical detail, and selection bias. Furthermore, the small sample size may limit the generalizability of our findings. Therefore, further prospective studies are necessary to analyze if the quality of care improves with MDT extremity boards in this challenging patient population.

## Conclusions

MDT meetings for patients with complex extremity defects offer a platform to formulate individual treatment plans and to avoid limb amputation in the majority of cases. Patient preferences and patient-related factors are crucial and must be considered to successfully implement the reached MDT meeting decision.

## Data Availability

All data is contained within the manuscript and other patient's details analysed during the current study available from the corresponding author on reasonable request.

## Abbreviations

MDT: Multidisciplinary team meeting
SD: Standard deviation
CI: Confidence interval
